# There Is No Impact of Diabetes on the Endothelial Function of Chronic Kidney Disease Patients

**DOI:** 10.1155/2018/7926473

**Published:** 2018-11-25

**Authors:** Mariana Nogueira Coutinho, Aluizio Barbosa Carvalho, Maria Aparecida Dalboni, Margaret Gori Mouro, Elisa Mieko Suemitsu Higa, Valéria Costa-Hong, Luiz Aparecido Bortolotto, Rejane Augusta de Oliveira Figueiredo, Maria Eugênia Fernandes Canziani

**Affiliations:** ^1^Federal University of São Paulo, Brazil; ^2^Heart Institute (InCor) of the University of São Paulo Medical School, Brazil; ^3^Folkälsan Research Center, Helsinki, Finland

## Abstract

**Background:**

Patients with chronic kidney disease (CKD) and type 2 diabetes mellitus (DM) have increased risk of endothelial dysfunction, cardiovascular disease, and mortality. Several studies have separately analyzed endothelial function in these populations. However, data of patients with both CKD and DM are scarce. The aim of this study was to evaluate whether the presence of DM has any additional effect on the endothelial dysfunction of CKD patients.

**Methods:**

We measured endothelial progenitor cells (EPCs), stromal-derived factor 1 alpha (SDF-1*α*), serum and urinary nitric oxide (NO), flow-mediated dilation (FMD), and pulse wave velocity (PWV) in 37 CKD patients with DM (CKD-DM group) and in 37 without DM (CKD group).

**Results:**

CKD-DM group had a higher prevalence of obesity (*P* < 0.01), previous myocardial infarction (*P* = 0.02), myocardial revascularization (*P* = 0.04), and a trend for more peripheral artery disease (*P* = 0.07). Additionally, CKD-DM group had higher EPC (*P* = 0.001) and PWV (*P* < 0.001) values. On the other hand, no difference in SDF-1*α* and serum or urinary NO and FMD was observed between the groups.

**Conclusions:**

Endothelial dysfunction is frequent in CKD patients, and an additive effect of diabetes cannot be implicated, suggesting the predominant role of uremia in this condition.

## 1. Introduction

The Global Burden Disease 2010 study had highlighted chronic kidney disease (CKD) as an important cause for global mortality [1]. It is estimated that 10–15% of the adult population has CKD at various stages of severity [2]. This rate has grown [3] in parallel with the increasing incidence and prevalence of type 2 diabetes mellitus (DM) [3, 4], one of the main causes of CKD [4].

It is well known that patients with CKD and DM have higher mortality rates compared to their counterparts without DM [2, 5]. Cardiovascular disease (CVD) is the most important cause of mortality in CKD as well as in DM patients [6, 7]. Endothelial dysfunction, the initial lesion of atherosclerosis [8, 9], is an early marker of CVD frequently observed both in CKD [10, 11] and DM patients [12]. Several factors are associated with endothelial dysfunction in these populations [13, 14], such as uremic toxins and hyperglycemia, that are related to the depletion of endothelial nitric oxide (NO) [12, 14–16]. Moreover, uremic toxins stimulate the expression of adhesion molecules, which are also associated with endothelial dysfunction [14].

The evaluation of endothelial function includes the measurement of endothelial progenitor cells (EPCs), which have been shown to take part in the endogenous vascular repair system. The EPC count is considered to be a predictor of endothelial dysfunction and cardiovascular outcomes [17, 18] in populations with known high cardiovascular risk, who have reduced number or impaired function of EPC. Several studies have been demonstrated that EPC number was reduced in patients with isolated CKD and DM, compared to healthy controls [19, 20]. Ozuyaman et al. [21] demonstrated that EPC mobilization and function require NO. Among other factors and chemokines, stromal cell-derived factor-1 alpha (SDF-1*α*) is the most potent chemokine that mobilizes EPC from bone marrow to the injured vessel sites [22, 23]. The levels of SDF-1*α* are associated with increased CVD risk, both in general [24] and CKD patients [25].

Endothelial dysfunction can also be quantified by the degree of flow-mediated dilation (FMD) of the brachial artery, a widely used noninvasive technique [26]. The reduction of FMD occurs early in the development of atherosclerosis [27]. Several studies have shown that FMD is impaired in CKD [28, 29] as well as in DM patients [20, 30].

Data regarding endothelial dysfunction in patients with concomitant DM and CKD is scarce. Therefore, we aimed to evaluate the impact of DM on the endothelial function of patients with CKD.

## 2. Materials and Methods

### 2.1. Study Subjects

In this case-control study, 74 patients with CKD were recruited: 37 patients with DM (CKD-DM group) and 37 patients without DM (CKD or control group), from the outpatients CKD clinic of the Federal University of São Paulo, Brazil.

The inclusion criteria were age ≥ 18 years and CKD stages 3a–4. Regarding diabetic patients, only those on insulin therapy were included. Exclusion criteria were type 1 or secondary forms of DM; use of oral hypoglycemic agents, erythropoietin, or estrogen supplementation; malignancy in the last 5 years; hepatic insufficiency, New York Heart Association class III/IV heart failure, acute myocardial infarction, or peripheral arterial disease, decompensated in the last 6 months; acute infectious disease in the last 30 days; and pregnant or breastfeeding and regular smokers. Regular smokers were considered to be those consumers of at least one daily cigarette for at least six months.

All patients underwent an assessment of their medical history, physical examination, laboratory tests, and endothelial evaluation, including EPC number, SDF-1*α*, serum and urinary NO levels, and FMD.

The study was reviewed and approved by the Ethics Advisory Committee of the Federal University of São Paulo (approval number: 569.458). All patients gave written informed consent.

### 2.2. Laboratory Tests

After a 12-hour overnight fast, blood samples were collected to measure serum creatinine, glucose, glycosylated hemoglobin (HbA1c), total HDL and LDL cholesterol, triglycerides, potassium, sodium, intact parathyroid hormone (iPTH—chemiluminescent microparticle immunoassay performed at ARCHITECT i4000, Abbott), ionized calcium, phosphate, alkaline phosphatase, bicarbonate, and blood count. Serum SDF-1*α* was determined by enzyme immunoassay (ELISA, Elabscience, Wuhan, Hubei, China). Nitric oxide was quantified in serum and 24-hour urine sample by chemiluminescence, using nitric oxide analyzer (NOATM 280, Sievers Instruments Inc., Boulder, CO, USA). Albuminuria was measured in 24-hour urine sample. The estimated glomerular filtration rate (eGFR) was calculated using the Chronic Kidney Disease Epidemiology Collaboration (CKD-EPI) equation.

### 2.3. Flow Cytometry Analysis of Circulating EPCs

**Figure 1:**
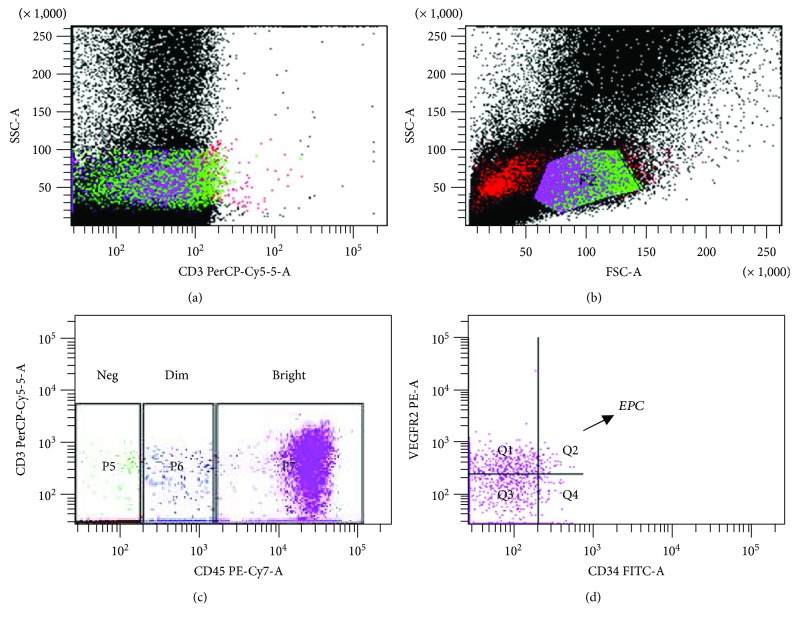
Analysis of EPC by flow cytometry: (a) labeling with CD3-PerCP for identification of lymphocytes, (b) lymphocytic gate, (c) identification of cells with CD45-dim, and (d) after identification of item (c), selection of those with labeling for CD34 and VEGFR2 (Q2).

Ten milliliters of peripheral blood was collected in an EDTA tube for EPC analysis. The blood samples were processed within 4 hours after collection. Mononuclear cells were separated using Ficoll–Hypaque density gradient centrifugation (Sigma-Aldrich, St. Louis, USA) and washed using phosphate-buffered saline (PBS) (Sigma-Aldrich, St. Louis, USA). An automatic cell counter was used to ensure that in each analyzed tube there were 1,000,000 cells. Subsequently, the samples were exposed to the following antibodies: CD45-PE-Cy7 (BD Biosciences, San Diego, USA), CD34-FITC (BD Biosciences, San Diego, USA), and VEGFR2-PE (BD Biosciences, San Diego, USA). Isotype-stained samples were used as negative control. After incubation in the dark, excess antibody was removed by washing with PBS. Lastly, the cells were washed with PBS buffered with sodium azide and analyzed by flow cytometry. To facilitate lymphocyte gate demarcation, CD3-PerCP or CD3-APC lymphocyte markers (BD Biosciences, San Diego, USA) were used in most samples. After demarcation of this gate, EPCs were identified by the low expression of CD45 and by CD45-dim and by the double expression of CD34 and VEGFR2 (Figure 1), as previously described [20, 31, 32].

The detection of all antibodies was performed by a flow cytometer (FacsCanto I, BD Biosciences, San Diego, CA, USA). The gated data of CD3^+^ (T lymphocytes), CD45-PE-Cy7, CD34-FITC, and VEGFR2PE were presented as cells per 900,000 events.

### 2.4. Measurement of Brachial Artery FMD

Ultrasound-based measurements of brachial artery reactivity were performed according to the guidelines of the International Brachial Artery Reactivity Task Force [33]. The assessment of vascular reactivity was always carried out by the same examiner who was blinded to the group allocation. The brachial artery was assessed and measured in longitudinal section just above the antecubital fossa using a high-resolution ultrasound system (Sequoia Echocardiography System, version 6.0, Acuson, Siemens, Vernon, CA, USA) equipped with a multifrequency linear transducer (7–12 MHz) to produce two-dimensional images. The techniques used to evaluate the changes of FMD and nitrate-mediated dilation (NMD) after physical and pharmacological stimulation, respectively, are described elsewhere [34].

### 2.5. Pulse Wave Velocity (PWV)

As a surrogate marker of subclinical atherosclerosis, arterial stiffness was noninvasively measured by the PWV of the carotid and femoral arteries. PWV was carried out by the same examiner who was blinded to the group allocation using the Complior SP equipment (Artech Medical, Pantin, France) and then analyzed by appropriate software.

### 2.6. Statistical Analysis

Mean and standard deviation, median, and interquartile range or frequencies (proportion) were calculated for each variable, as appropriate. The Kolmogorov-Smirnov statistical test was used to investigate the normal distribution of data. Comparisons of continuous variables were performed using Student's *t*-test and the Mann-Whitney *U* test, for normal and skewed data, respectively. Comparisons of proportions were performed using chi-squared analysis or Fisher's exact test, as appropriate. Among the variables that evaluated endothelial function, FMD was the only one that showed normal distribution. Thus, generalized linear models (GLMs) were performed with normal or gamma distribution, according to the variable characteristics. For the assessment of FMD, the model was adjusted to the following variables: age, gender, peripheral artery disease, and use of acetylsalicylic acid and antihypertensive drugs; for the evaluation of SDF-1*α*: age, gender, and use of acetylsalicylic acid; and for the EPC assessment: use of acetylsalicylic acid, lipid-lowering agents, and CD3 type. *P* values < 0.05 were considered statistically significant. All statistical analysis was performed using the SPSS for Windows (SPSS 19.0, Chicago, IL, USA). The sample size was calculated based on previous study by Wong et al. [35]. For this calculation, a conservative approach was adopted and was performed using the Gpower 3.1.2 software. Assuming a difference in EPCs and FMD of 50%, a total of 74 subjects, 37 in each group, were required to reach a level of significance of 5% and a power of 80%.

## 3. Results

Characteristics of the CKD patients according to the presence (CKD-DM group) or absence (CKD group) of diabetes are listed in Tables 1 and 2. There was a predominance of elderly hypertensive men in both groups.

**Figure 2:**
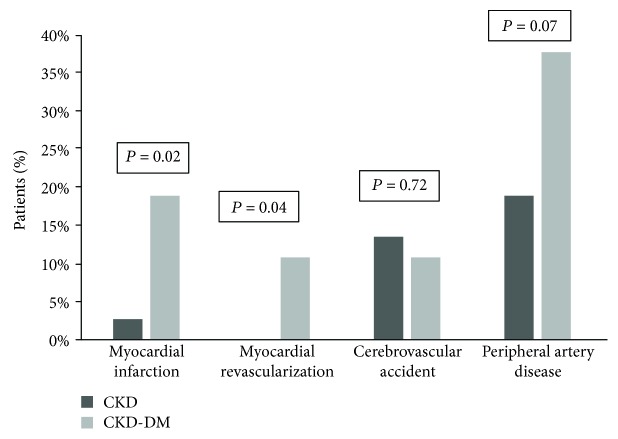
Previous cardiovascular disease according to the groups.

When compared to the CKD group, patients with CKD-DM showed a higher prevalence of previous myocardial infarction, myocardial revascularization, and a trend for more peripheral artery disease (Figure 2). Diabetic patients received more diuretic and acetylsalicylic acid but less calcium channel blocker. There was no difference in the use of ACEI/ARB or lipid-lowering drugs. The CKD-DM group had higher proportion of obese individuals (21 (57%) vs. 9 (24%); *P* = 0.004). Of note, only 2 patients in the CKD group had waist-hip circumference ratio within normal range. A higher prevalence of patient with uncontrolled systolic blood pressure was observed among CKD-DM patients (20 (54) vs. 11 (30) %; *P* = 0.034).

**Figure 3:**
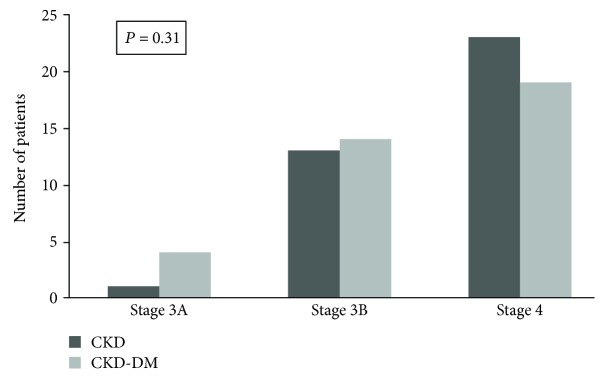
Distribution of patients based on stages of CKD.

Renal function as well as the distribution of patients according to CKD stages did not differ between groups (Figure 3). As expected, the CKD-DM group had higher albuminuria, glucose, HbA1c, and triglyceride levels. There was a trend towards lower HDL in this group. Bicarbonate was significantly higher in the CKD-DM group, although the supplementation of bicarbonate was similar in both groups. Alkaline phosphatase was significantly higher in the CKD-DM group; however, only 3 patients (2 of CKD-DM group) presented serum levels above the normal range. There was no difference in hemoglobin (Hb) concentration between the groups, and all patients had Hb greater than 10 mg/dl. The PWV was higher in the CKD-DM group as well as the proportion of patients with increased values (21 (60) vs. 8 (22) %; *P* = 0.001).

EPC number was higher in the CKD-DM group compared to CKD. The FMD was similar, showing low values in both groups. Of note, 10% of the patients in each group failed to display any dilation during the test. No difference in SDF-1*α* and serum or urinary NO between the groups was observed (Table 3).

When the sample was divided based on CKD stages, there was no difference in endothelial parameters (EPC, SDF-1*α*, serum, and urinary NO and FMD) (Table 4).

## 4. Discussion

The present study has demonstrated a high prevalence of endothelium dysfunction in CKD patients regardless the presence of diabetes. All the endothelial dysfunction markers, but EPC number, were similar in CKD patients with and without diabetes.

Few studies, including dialysis and nondialysis patients, demonstrated a similar number of EPCs in CKD patients with and without DM [36–38]. In contrast with these studies, our results showed that CKD-DM patients had higher EPC number. This unexpected finding could be related to the fact that all diabetic patients were using insulin, which is known to increase the EPC number [39, 40]. Moreover, one could hypothesize that this elevated number of EPC reflects a better activity of the endogenous vascular repair system. However, our CKD-DM patients had high prevalence of cardiovascular disease and an inadequate arterial stiffness, revealed by the increased PWV. Based on that, we could speculate that the EPC could be dysfunctional or the increased number might be insufficient to repair the vessels.

It is well known that diabetic patients have EPC dysfunction [20, 41] mainly due to hyperglycemia [41, 42]. High glucose level leads to an increasing of advanced glycation end products, reactive oxygen species, and inflammatory cytokines, factors that could induce EPC dysfunction [12, 20, 43]. Likewise, there are substantial data indicating EPC dysfunction in CKD patients [16, 44]. Uremic environment causes a deficient NO production, which leads to a decreased EPC mobilization [38]. Additionally, uremic toxins were found to cause EPC dysfunction by inhibiting migratory activity, adhesion to matrix proteins and to endothelial cells [38, 45]. Corroborating with that, studies have suggested that the reduction of uremic toxins by kidney transplantation improves EPC function [46, 47]. Unfortunately, we did not evaluate EPC function in the present study.

Other factor that could be related to the endothelial dysfunction observed in our CKD patients is the EPC resistance. Herbrig et al. observed that chronically elevated SDF-1*α* levels result in impair EPC homing to sites of vascular damage, indicating EPC resistance [46]. Of note, Jie et al. demonstrated that SDF-1*α* is increased in CKD patients due to its reduced renal clearance [19]. On the other hand, in diabetic patients, SDF-1*α* concentration has been shown to be decreased. The diminished concentration of SDF-1*α*, observed in that diabetic population, was associated to the reduction of EPC releasing to the damaged vessels [48]. In the present study, SDF-1*α* levels were high in both CKD and CKD-DM groups, which may suggest that uremia effect on SDF-1*α* overcomes that of diabetes. Supporting this hypothesis, serum and urinary NO and FMD values were similar in both groups. In agreement with this finding, previous studies, including nondialysis [49] and dialysis [50] patients, were not able to demonstrate that the presence of diabetes had influenced FMD, even after adjustments for confounding factors.

Some limitations of this study should be acknowledged, such as the relative small sample size, the absence of a healthy control group, and its cross-sectional design. Nevertheless, to the best of our knowledge, this is the first study designed to investigate the additive effect of diabetes on the endothelial function of CKD patients.

## 5. Conclusion

Endothelial dysfunction is frequent in CKD patients, and an additive effect of diabetes cannot be implicated, suggesting the predominant role of uremia in this condition.

## Figures and Tables

**Table 1:** Clinic characteristics of the study population.

	CKD group (*n* = 37)	CKD-DM group (*n* = 37)	*P*
Age, years	65.9 ± 13.9	64.1 ± 9.9	0.54
Male, *n* (%)	21 (56.8%)	22 (59.5%)	0.814
Hypertension, *n* (%)	35 (97.2%)	34 (94.4%)	0.555
Chronic kidney disease etiology, *n* (%)			<0.001
Diabetes	0 (0%)	33 (89.2%)	
Undetermined	19 (51.4%)	1 (2.7%)	
Hypertension	7 (18.9%)	0	
Glomerulopathy	5 (13.5%)	0	
Others	6 (16.2%)	3 (8.1%)	
Cardiovascular disease, *n* (%)	12 (32.4%)	19 (51.4%)	0.099
Myocardial infarction	1 (2.7%)	7 (18.9%)	0.025
Myocardial revascularization	0 (0%)	4 (10.8%)	0.04
Cerebrovascular accident	5 (13.5%)	4 (10.8%)	0.722
Peripheral artery disease	7 (18.9%)	14 (37.8%)	0.071
Drugs, *n* (%)			
ACEI/ARB	27 (75%)	33 (91.7%)	0.058
Calcium channel blockers	27 (75%)	17 (47.2%)	0.016
Diuretics	25 (69.4%)	34 (94.4%)	0.006
Vasodilator	2 (5.6%)	7 (19.4%)	0.075
Lipid-lowering agents	24 (68.6%)	30 (85.7%)	0.088
Acetylsalicylic acid	14 (38.9%)	27 (73%)	0.003
Systolic blood pressure, mmHg	130 (125–140)	140 (123.5–158.5)	0.214
Diastolic blood pressure, mmHg	80 (70–90)	77 (70–84)	0.343
Body mass index, kg/m^2^	27.7 ± 4.7	31.4 ± 5.7	0.004
Waist-hip circumference ratio	0.97 ± 0.07	1.00 ± 0.06	0.032

ACEI = angiotensin-converting enzyme inhibitor; ARB = angiotensin receptor blocker.

**Table 2:** Laboratorial characteristics of the study population.

	CKD group (*n* = 37)	CKD-DM group (*n* = 37)	*P*
Creatinine, mg/dl	2.33 ± 0.65	2.22 ± 0.65	0.442
CKD-EPI, ml/min/1.73 m^2^	24 (21–34.5)	28 (23.5–35.5)	0.267
Albuminuria, *μ*g/min (24 h)	42.1 (11.5–131.7)	132.3 (39.5–767.4)	0.014
Glucose, mg/dl	88 (85–92)	142 (80–206)	0.003
Hemoglobin A1c, %	5.6 (5.3–5.9)	8.4 (7.2–9.9)	<0.001
Total cholesterol, mg/dl	163 (151–187)	183 (141–217)	0.141
HDL cholesterol, mg/dl	52 (42–61)	46 (40–53)	0.074
LDL cholesterol, mg/dl	90 (70–105)	103 (71–123)	0.113
Triglycerides, mg/dl	128 (90–183)	176 (126–305)	0.002
Ionized calcium, mmol/l	1.31 ± 0.06	1.29 ± 0.06	0.095
Phosphate, mg/dl	3.5 ± 0.6	3.6 ± 0.6	0.338
Alkaline phosphatase, U/l	67 (60–86)	80 (66–95)	0.036
Bicarbonate, mmol/l	24.8 ± 2.8	26.9 ± 3.4	0.005
Parathyroid hormone, pg/ml	163 (96–240)	167 (117–210)	0.948
Hemoglobin, g/dl	13.5 ± 1.6	13.7 ± 1.6	0.607
Pulse wave velocity, m/s	8.5 ± 1.8	10.3 ± 1.7	<0.001

HDL = high-density lipoprotein; LDL = low-density lipoprotein.

**Table 3:** Endothelial dysfunction markers in CKD and CKD-DM groups.

	CKD group (*n* = 37)	CKD-DM group (*n* = 37)	*P*
FMD, %	2.68 ± 3.11	2.95 ± 3.69	0.737
NMD, %	11.51 ± 6.05	9.26 ± 5.47	0.104
EPC, %	0.25 (0.1–0.6)	0.60 (0.3–0.9)	0.009
SDF-1*α*, pg/ml	3730 (2915–4830)	3430 (2695–4770)	0.699
Serum nitric oxide, *μ*mol/l	390.5 (296.5–568.8)	387.5 (241.5–613.8)	0.641
24 h urinary nitric oxide, *μ*mol	3432 (1593–5521)	3336 (1213–5896)	0.734

FMD = flow-mediated dilation; NMD = nitrate-mediated dilation; EPC = endothelial progenitor cell; SDF-1*α* = stromal cell-derived factor 1.

**Table 4:** Endothelial dysfunction markers based on CKD stages.

	CKD 3	CKD 4	*P*
FMD, %	2.78 ± 3.26	2.85 ± 3.55	0.930
EPC, %	0.4 (0.2–1.0)	0.3 (0.1–0.7)	0.180
SDF-1*α*, pg/ml	3535 (2935–4927)	3800 (2697–4472)	0.739
Serum nitric oxide, *μ*mol/l	414.0 (294.0–581.2)	347.0 (260.0–586.9)	0.659
24 h urinary nitric oxide, *μ*mol	3121 (2224–6348)	3149 (1010–4976)	0.252

CKD 3 = chronic kidney disease stage 3; CKD 4 = chronic kidney disease stage 4; FMD = flow-mediated dilation; EPC = endothelial progenitor cell; SDF-1*α* = stromal cell-derived factor 1.

## Data Availability

The data used to support the findings of this study are available from the corresponding author upon request.
